# Adaptation Difficulties of Children at the Beginning of School Attendance Based on the Optics of Primary School Teachers

**DOI:** 10.3390/children10020410

**Published:** 2023-02-20

**Authors:** Radka Teleková, Tatiana Marcineková, Anna Tirpáková, Dalibor Gonda

**Affiliations:** 1 Department of Pedagogy, Faculty of Education, Constantine the Philosopher University in Nitra, Dražovská 4, 949 01 Nitra, Slovakia; 2 Department of Mathematics, Faculty of Natural Sciences, Tr. A. Hlinku 1, Constantine the Philosopher University in Nitra, 949 01 Nitra, Slovakia; 3 Department of School Education, Faculty of Humanities, Tomas Bata University in Zlín, Štefánikova 5670, 760 00 Zlín, Czech Republic; 4 Department of Mathematical Methods and Operations Research, Faculty of Management Science and Informatics, University of Žilina, Univerzitná 1, 010 01 Žilina, Slovakia

**Keywords:** adaptation difficulties, beginning of schooling, pupils, teachers

## Abstract

The presented paper is devoted to finding out and analyzing the opinions of primary education teachers on the causes of the unsuccessful adaptation of current schoolchildren to the beginning of systematic education. To find out the above issues, pedagogical research was carried out at selected primary schools in Slovakia. The implementation of the research and the subsequent analysis of the research results confirmed that the length of teachers’ pedagogical practice has a statistically significant effect on their views on the causes of adaptation difficulties in emotional, social, intellectual, and psychomotor areas of children’s school readiness.

## 1. Introduction

By accepting the first socially obligatory role of a pupil, the child enters new educational conditions, to which he/she gradually adapts. In our case, it is a process of school adaptation. Some authors define it as an active adaptation of the individual to the social environment of the school and the school per pupil [1]. Understanding the individual aspects of the school adaptation process and creating optimal conditions from all concerned will help many children to facilitate its course and eliminate or alleviate the emerging adaptation difficulties. Helping children to cope with the adaptation process is very important, because failure to manage this process can also manifest itself in the form of the deteriorating health of the child [2].

The causes of pupils’ adaptation difficulties to the school environment can be divided into internal and external [1,3,4,5]. Internal causes are mainly conditioned by the child’s personality characteristics, school immaturity (immaturity of individual brain structures, underdeveloped neuropsychic functions), low levels of communicative skills, and inability to work constructively with teachers and classmates [6]. Some authors [7,8,9,10,11] consider the lower level of individual areas (socio-emotional area, intellectual area, psychomotor area) of school readiness to be a source of potential causes of adaptation difficulties in children.

Gradual identification with the role of a pupil is associated with an increased number of new social contacts with new classmates and teachers, which requires the regulation of one’s own social space. This process is a source of other internal adaptation problems. Pupils were identified as having insufficient orientation to the teacher’s requirements and no respect for the rules of conduct [5] inability to create an acceptable position in the classroom [6,12] unpopularity among classmates, and the inability to form satisfactory relationships and contacts with peers [13,14] and finding a suitable position in the group [15,16,17].

The external causes of the pupil’s adaptation difficulties are conditioned by the different natures of the educational process; in the primary school, the teaching process has a more formal character compared to pre-primary education. New rules and requirements that determine the daily activities of pupils come to the fore. [18,19,20].

Smooth acceptance and coping with all changes affect the quality of the adaptation process, which has an impact on the academic success of children not only at the beginning of school, but also in the future. During their research, [21] concluded that children with better adaptability were able to adapt more flexibly to new life situations later in life, were more satisfied, and showed more social commitment compared to children with lower levels of adaptability. At the same time, the success of adaptation also affects the healthy development of the child’s personality [22,23].

Based on the above research, there is still a current need to find effective strategies to support the successful adaptation of children to the school environment [24,25,26,27,28,29] from kindergarten and family to the first year of primary school, which is considered one of the critical periods in a child’s life [30]. The effectiveness of this process will depend on the level of school readiness of the child and his/her parents, as well as on the readiness of the teacher and the school. The ability to understand the experiences and behavior of children requires a certain level of sensitivity to the difficulties of children, the ability to recognize their manifestations and causes, and in this context, the choice of appropriate strategies to support the child and his family.

## 2. Material and Methods

The effectiveness of the adaptation process will depend on the level of school readiness of the child and his/her parents and finally on the readiness of the teacher and the school. According to [31] teachers with longer experience have more experience in teaching different groups of children entering the first year of primary school, so it can be assumed that they will be able to detect signals of pupils’ adaptation difficulties in time. The diagnostic competencies of the teacher also play an important role in this. The results of the research by [32] show that the results of pedagogical diagnostics of teachers in this area are comparable with the findings of psychiatrists and psychologists, i.e., teachers can correctly diagnose the manifestations and causes of children’s adaptation difficulties. Thus, we can assume that for a beginning teacher, the starting point of working with pupils is mainly the theoretical knowledge acquired during the study, and the teacher with a longer experience mainly uses the acquired experience.

Theoretical background and research findings motivated us to carry out pedagogical research, the aim of which was to find out and analyze the attitudes and opinions of primary education teachers on the causes of adaptation difficulties of current freshmen and on the possibilities of their prevention or elimination.

We carried out pedagogical research at the beginning of the 2020/2021 school year. The research sample consisted of a total 104 primary school teachers from all over Slovakia, who were categorized according to the length of teaching experience into five groups: teachers with up to 5 years of experience, with teaching experience of 5 to 10 years (inclusive), 10 to 15 years (inclusive), 16 to 20 years of teaching experience (inclusive), and teachers with more than 20 years of experience.

We chose an electronic questionnaire of our own design as the main research tool for finding out the opinions of teachers (see Appendix A). The author’s questionnaire had 16 closed questions, i.e., with multiple choice. Respondents were able to indicate the selected number of answers from the options offered. We verified the validity and reliability of the compiled questionnaire using Cronbach’s alpha test. The calculated value of 0.91 confirmed the validity of the questionnaire.

The questionnaire contained questions aimed at finding out the respondents’ opinions on the causes of adaptation difficulties, specifically in the following four areas of children’s school readiness: in the emotional, social, intellectual, and psychomotor areas. Each area contained five questions. In all four areas of school readiness, respondents could answer “yes” or “no” to the questions. Subsequently, for each question, we evaluated the number of positive, and negative answers, respectively. We analyzed the obtained results with respect to the length of teachers’ pedagogical practice. Selected statistical methods were used in the analysis.

In compiling the questions, we were inspired by the authors [5,24] who dealt with the issue of potential “risk factors” for the failure of children to adapt to new school conditions. The author [24] defines the factors influencing pupil adaptation at the beginning of schooling, such as the social–emotional and communication skills of children, stimulating the family environment, and the completion of pre-primary education. The acquired experience of the child from kindergarten (day mode, contact with teachers, peers, guided activities) is a prerequisite for a more successful adaptation to the new environment. In research by authors [5] identified potential reasons for adaptation difficulties for beginning schoolchildren, e.g., insufficient orientation to the requirements of the teacher, no respect for the rules of conduct, and no respect for the school regime.

## 3. Analysis and Interpretation of the Results of the Questionnaire for Teachers

Before starting the research, we set the following research hypothesis:

H1: The length of practice of primary school teachers has an impact on their views and attitudes on the causes of adaptation difficulties in first-year pupils.

To verify the validity of the established hypothesis, we analyzed the answers obtained to all questions of the questionnaire, which concerned the opinions and attitudes of teachers on the causes of children’s adaptation difficulties in the emotional, social, intellectual, and psychomotor areas.

We analyzed the validity of the research hypothesis using statistical methods. The aim of the statistical analysis was to find out whether there is a connection between the length of primary school teachers’ practice (five groups of teachers, created according to the length of their internship) and their views on the causes of adaptation difficulties of first-year pupils.

In the statistical analysis of the obtained data, we used selected test methods, namely the $\chi^{2}$ independence test for the contingency table type k×m [33].

In the statistical analysis, we were interested in the existence of statistically significant connections between respondents ‘views on the causes of adaptation difficulties of first-year pupils in all four areas of school readiness: emotional, social, intellectual and psychomotor, and the length of the teachers’ practice.

(A) Causes of adaptation difficulties in the emotional area of school readiness

In examining the causes of adaptation difficulties in the emotional area of school readiness, we tested—with the $\chi^{2}$—test whether there is a statistically significant relationship between the length of teachers ‘practice and teachers’ answers to all questions from the emotional area. In the case of proven statistical significance of the relationship between the length of teachers’ practice and their answers to a given question from the emotional area, we also calculated the value of the contingency coefficient. The results are shown in Table 1.

From Table 1, we see that we reject the null hypothesis $H_{0}$ about the independence of the observed features at the level of significance α=0.01 and accept the alternative hypothesis $H_{1}$ in two cases (questions 1 and 2). In other cases (questions 3–5), we cannot reject the null hypothesis $H_{0}$ about the independence of the observed features. This means that the length of the teacher’s practice has a statistically significant effect on whether the insufficient school motivation, easy distraction of the pupil by external stimuli, can be considered the cause of adaptation difficulties. Based on the results obtained using statistical methods, we state that the length of the teachers’ practice does not have a statistically significant effect on the answers to questions no.3, 4, and 5. This means that teachers do not consider the need for verbal support and encouraging communication, physical proximity, and constant adult contact, as well as rejection, anger, and aggression, as the cause of adaptation difficulties in 1st grade pupils.

**Figure 1:**
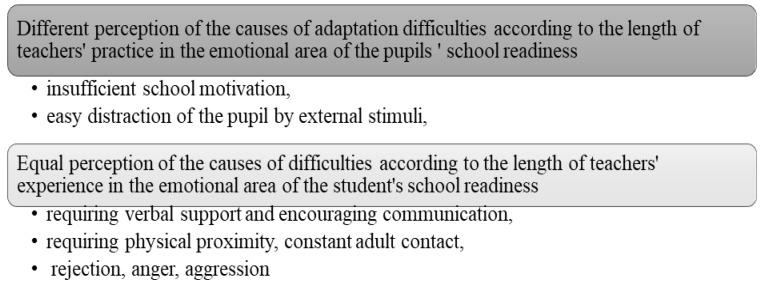
Perception of the causes of adaptation difficulties according to the length practice.

After calculating the value of the contingency coefficient between the identification of the causes of adaptation difficulties in first-year pupils and the length of the teacher’s practice, we found a high degree of binding in both cases, i.e., there is a high degree of attachment between the length of a teacher’s practice and the ability to identify insufficient school motivation (Figure 1).

Teachers with shorter experience do not perceive insufficient school motivation as the cause of pupils’ adaptation difficulties, but according to teachers with longer experience, it is one of its possible causes. Moreover, teachers with shorter experience do not perceive the pupils being easily distracted by external stimuli as the cause of pupils’ adaptation difficulties, but on the contrary, teachers with longer experience do.

Teachers, regardless of the length of their internship, perceive the requirement of physical proximity, constant adult contact, as the cause of adaptation difficulties. On the other hand, they do not perceive the requirement of verbal support and encouraging communication, as well as rejection, anger, and aggression, as the cause of adaptation difficulties in the emotional area.

(B) Causes of adaptation difficulties in the social area of school readiness

Analogously, both in the emotional area and in the social area, we tested whether there was a statistically significant relationship between the length of teachers ‘practice and teachers’ answers to all social questions. We also calculated the value of the contingency coefficient in the case of proven statistical significance of the relationship between the length of teachers’ practice and their answers to a given social question. The results are shown in Table 2.

From Table 2, we can see that we reject the null hypothesis $H_{0}$ of independence of the observed signs at the level of significance α=0.01 and accept the alternative hypothesis $H_{1}$ in three cases (questions 1, 2, and 4), and in two cases (questions 3 and 5), the null hypothesis $H_{0}$ of independence of observed characters cannot be rejected.

**Figure 2:**
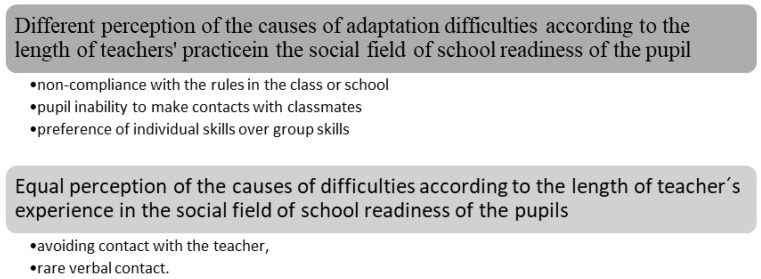
Perception of the causes of adaptation difficulties in the social field.

Based on the calculated values of the contingency coefficient, there is a high degree of connection between the length of teaching practice and the identification of non-compliance in the classroom or school. A very close link was identified between the length of the internship and the identification of the pupils’ inability to contact classmates. There is also a very close link between the length of teaching practice and the perception of the preference of individual activities over group activities as causes of adaptation difficulties (Figure 2).

Teachers with shorter experience do not consider non-compliance with the rules in the classroom or at school, the inability of the pupil to establish contacts with classmates, or the preference of individual activities over group ones due to adaptation difficulties, while teachers with longer experience do.

Teacher avoidance and rare verbal contact are not considered by teachers to be adaptation problems, regardless of the length of the internship.

(C) Causes of adaptation difficulties in the intellectual field of school readiness

In the intellectual field, we used a chi-square test to test the statistical significance of the relationship between the length of teachers’ experience and their answers to questions from the intellectual field of school readiness. We also calculated the value of the contingency coefficient in the case of proven statistical significance of the relationship between the length of teachers’ practice and their answers to a given intellectual question. The test results are shown in Table 3.

**Figure 3:**
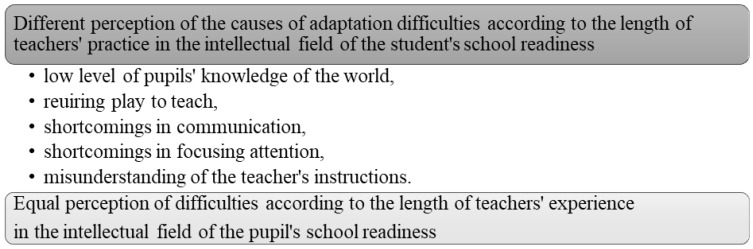
Perception of the causes of intellectual adaptation difficulties.

Table 3 shows that the answers to all questions are statistically significantly influenced by the length of teachers’ experience. The calculated values of the contingency coefficients in all cases represent a high degree of binding (Figure 3).

Teachers with shorter internships consider pupils’ low level of knowledge of the world and the requirement to play in school as causes of adaptation difficulties, and the other items shown in Figure XY do not cause adaptation difficulties. Teachers with longer experience spoke in the opposite way about all items.

(D) Causes of adaptation difficulties in the psychomotor area of school readiness

As in the previous areas, we also tested, with the $\chi^{2}$ test, the statistical significance of the relationship between the length of teachers’ practice and their answers to questions from the psychomotor area of school readiness. We also calculated the value of the contingency coefficient in the case of proven statistical significance of the connection between the length of teachers’ practice and their answers to a given question from the psychomotor area. The test results are shown in Table 4.

**Figure 4:**
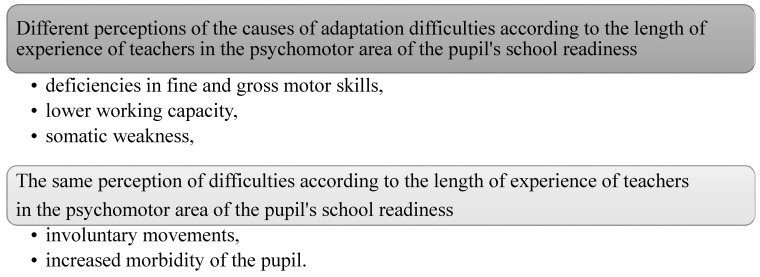
Perception of the causes of adaptation difficulties in the emotional area.

From Table 4 we can see that we reject the null hypothesis $H_{0}$_0_ about the independence of the observed signs at the level of significance α=0.01 and accept the alternative hypothesis $H_{1}$ in two cases (questions 1 and 4), and in one case (question 5), we also reject the null hypothesis $H_{0}$ in favor of the alternative hypothesis $H_{1}$, but at the level of significance α=0.05. Based on the values of the contingency coefficients given in Table 3, we state that there is a very close link between the length of a teacher’s experience and the identification of deficiencies in fine or gross motor skills as the cause of adaptation difficulties. The calculated value between the length of the teacher’s experience and the identification of lower working capacity, also by somatic weakness as the cause of adaptation difficulties, indicates a high degree of attachment (Figure 4).

In the psychomotor field, teachers with shorter experience perceive deficiencies in fine and gross motor skills and somatic impairment as causes of adaptation difficulties, but teachers with longer experience do not. With the item lower working capacity, it is the opposite. Only teachers with longer experience perceive it as the cause of adaptation difficulties. The items involuntary movements and increased illness of the pupil are not considered by teachers as causes of difficulties, regardless of the length of practice.

## 4. Discussion

Based on the results obtained by the questionnaire method, we can conclude that using statistical methods and subsequent analysis of research results, we managed to demonstrate a statistically significant relationship between the length of primary school teachers and some of their attitudes and views on adaptation problems of first-year pupils.

Similarly, Güner & Kartal [31] found that teachers with longer internships have more experience teaching different groups of beginning schoolchildren, which suggests their ability to identify signals of pupils’ adaptation difficulties in a timely manner. The author [32] also proves in his research that the findings from the diagnostic activity of teachers are in most cases comparable with the findings of psychiatrists and psychologists.

We further analyzed the same perception of the causes of adaptation difficulties according to whether they unanimously consider the given cause to be the cause of adaptation difficulties. Teachers with different lengths of practice also consider the requirement of physical proximity, constant contact of an adult, to be the cause of adaptation difficulties. Teachers with different lengths of practice also do not consider the need for verbal support and encouraging communication to be the cause of adaptation difficulties; rejection, anger, and aggression; avoiding contact with the teacher; rare verbal contact; involuntary movements; and increased illness of the pupil.

We found that teachers with longer experience consider their lack of preparation in the social–emotional, intellectual, and psychomotor areas to be the most common causes of children’s adaptation difficulties.

In the emotional area, we found a statistically significant relationship between the length of the pedagogical practice of respondents and the identification of the causes of adaptation difficulties, specifically in insufficient school motivation, a low level of ability to concentrate (easy distraction from external stimuli). We can state that almost twice as many teachers with more than 20 years of experience consider school motivation to be the cause of difficulties for pupils in the period of adaptation compared to that with teachers with less than 5 years of experience.

Through research findings, we draw attention to the need to develop children’s school motivation, as its low level is one of the important determinants of adaptation difficulties. We supplement our results with similar findings from a research study in Turkey, where K. Cokuka-I. Kozikoglu [34] classify low interest in school education and the level of motivation to school as the causes of difficult adaptation of the pupil. The authors [35] condition the success of adaptation to initial teaching by the level of positive survival. In a Danish study, S. Bronström [36] found that 12% of the children in the research sample showed nervousness and uncertainty associated with the expected start of schooling. Thus, the perception of school motivation as a cause of adaptation difficulties by teachers with experience of up to 5 years does not coincide with the theoretical basis, which corresponds to the perception of teachers with experience of more than 20 years.

M. Havlíková [37] points to the growing number of insufficiently motivated children to work in school, who subsequently fail in it and experience the burden. She mentions reasons, such as cognitive burden (attention, memory, thinking), so pupils quickly focus on less-demanding stimuli compared to learning activities. The theory is shared by teachers with more than 20 years of experience, according to which easy distraction with external stimuli is the cause of adaptation difficulties. However, teachers with less than 5 years of experience do not perceive this.

In the social field of school readiness of children, our respondents with more than 20 years of experience agreed (more than 25% of respondents), compared to teachers with shorter experience (5–14% of respondents), on the existence of causes of adaptation difficulties caused by non-compliance with class rules and the pupils’ inability to contact peers.

Following these findings, several authors [5,13,15] point to the need to form a relationship and contact with peers, which later has an impact on the adaptation of children. In an Australian research study, two experts [27] B. Perry and S. Dockett (2003) similarly emphasize the need for the socio-emotional development of pupils. Since, according to teachers with less than 5 years of experience, the inability to make contact with classmates and avoiding contact with teachers is not the cause of adaptation difficulties, their perception is at odds with the theoretical basis. Even in the case of non-compliance with the rules in the classroom and school, the opinions of teachers with less than 5 years of experience do not agree with the findings of experts, such as V. V. Gagay and Y.K. In their research study, ref. [5] identified the phenomenon as the cause of adaptation difficulties. We can consider the perception of the causes of adaptation difficulties in the social field by teachers with more than 20 years of experience to be in line with theoretical and research findings.

From the results of our research, we also found that in the intellectual field, there is a statistically significant relationship between the answers to all the questions asked and the length of pedagogical practice of the respondents. According to our findings, the perception of the causes of adaptation difficulties in the intellectual field is conditioned by the teacher’s practice. We note that in all cases, teachers perceived the causes of adaptation difficulties differently.

In analyzing the answers to the first question, which concerned pupils’ low level of knowledge of the world, i.e., knowledge about school, about learning activities, about man and his/her activities, about nature, about art, and knowledge about social norms as one of the reasons for the longer course of adaptation, this was more often mentioned by teachers with experience up to 5 years. Positive answers prevailed. Teachers with longer experience answered “no” more often. Following our further findings, these teachers pay more attention to the level of socio-emotional development than the teachers in the first group. These findings can be supported by a research study by the authors [38] who state that teachers and parents often focus on the necessary knowledge and understanding of the pupil, while underestimating the habits and skills for school education. To put this statement in perspective, it should be noted that a certain level of knowledge of the beginning schoolchildren about the world is necessary, as it is a basic basis of children’s future knowledge of objective reality, which is followed by the content of individual educational areas in the 1st year and beyond and training.

At the beginning of schooling, there is a change in the dominant activity, which becomes a learning activity. In kindergarten, play prevailed as a tool for developing a child’s personality, acquiring new knowledge and skills. The authors [39,40,41] state that in the context of the transition to primary school, it is a change of the game to learn according to the instructions of others, from spontaneous to teacher-driven activities. According to the author [8] after entering the 1st grade, pupils experience difficulties caused by playing, a disturbance based on the immature behavior of pupils.

In this case, similar opinions were expressed by teachers with less than 5 years of experience, who unlike teachers with more than 20 years of experience, consider the requirement of play to be the cause of adaptation difficulties.

According to our research findings about pupils’ shortcomings in communication as the cause of adaptation difficulties, teachers with less than 5 years of experience and teachers with 20 or more years of experience expressed the opposite. According to the shortest trained teachers, these shortcomings are not among the causes of difficulties (20% of respondents), and conversely, the teachers with the longest experience consider them to be common causes (23%). According to teachers with more than 20 years of experience, the perception of communication deficiencies as the causes of adaptation difficulties corresponds to current research results and theoretical approaches.

M. Yüksel et al. [42] state that there are communication problems for beginning schoolchildren, which relate to other difficulties, such as closedness, passivity, and a lack of cooperation in the team. In the professional literature [3,5] the internal characteristics of the child are mentioned, which condition individual adaptation at the beginning of schooling. Experts consider the insufficient mental maturity of children, the low level of their cognitive development, and preconditions for learning activities to the stated causes of maladaptation. Similar findings are noted by the authors [6] to them, the dominant factor in the maladaptation of children is the low level of their communication skills.

N. N. Tarasenko and M. L. Kovalenko [7] consider lack of focus on mental functions the cause of difficulties. L. Valentová [9] also complements the reduced concentration of attention. Although the theoretical framework of the issue confirms that shortcomings in the focus of attention are among the potential causes of difficulties, teachers with less than five internships do not perceive this. In agreement with the theory, teachers with more than 20 years of experience spoke.

The level of psychomotor development of the future pupil is one of the other assessed indicators before the child enters the 1st grade. In this area, as well, we have been able to demonstrate statistically significant correlations between the length of teaching experience and the answers to questions concerning shortcomings in fine and gross motor skills, lower working capacity.

When comparing and analyzing the answers, twice the number of positive answers was recorded for teachers with 20 years of experience and more than those for teachers with a length of practice from 1–5. Although the identification of deficiencies in the psychomotor development of the child is mainly based on the diagnostic competence of the doctor, the research results show the ability of teachers to diagnose related difficulties depending on the length of practice. Teachers with longer experience were better able to identify these causes than teachers with shorter teaching experience.

Similar results are reported by the authors [34,43] who in a research study found insufficient development of fine motor skills as a potential reason for difficulties in the period of adaptation. The finding is in line with the perception of teachers with more than 20 years of experience, who consider shortcomings in fine and gross motor skills to be the cause of adaptation difficulties.

The burden to which students are exposed in the initial teaching can also cause physical problems or even psychosomatic illness [44,45] advises among the potential causes of low levels of work and attention of children, which is made visible by exhaustion and fatigue. These theoretical starting points correspond to the perception of the cause by teachers with more than 20 years of experience. In practice, these are often visible phenomena that require attention to successfully manage the adaptation process. J. B. Vilčinskaja [43] also considers the causes of adaptation difficulties in the given area as shortcomings in fine or gross motor skills and somatic weakening of the child, which we also found in the perception of teachers with less than 5 years of experience.

## 5. Conclusions

Through the implementation of our pedagogical research, we concluded that if we want to eliminate or prevent various difficulties of children in the transition from intimate family and kindergarten to the formal environment of primary school, it is necessary to know the specifics of the educational process at the beginning of attendance, manifestations and causes of children’s difficulties, and appropriate strategies for their elimination or mitigation.

Based on the results of the research, we can also state that teachers can forecast the adaptation problems mostly correctly. This means that their findings are often in line with the findings of psychologists, psychiatrists, and other experts in the field. It was confirmed that a necessary condition for the successful transition of children to primary school is to ensure continuity between pre-primary and primary education. A related prerequisite is the active participation of teachers from both levels of education and parents, with a focus on stimulating individual areas of school readiness of future schoolchildren. Emphasis is placed primarily on the development of the personality component, which presupposes the development of individual–psychological characteristics of the child’s personality, his/her motivational sphere, preconditions for learning activities, and communicative competences in accordance with the individual possibilities and abilities of each child.

## Figures and Tables

**Table 1:** Results of $\chi^{2}$ test.

Question	$\chi^{2}$	*p*	*C*
1	18.781	0.000 *	0.88
2	16.641	0.002 *	0.85
3	1.09151	0.895	-
4	4.91480	0.296	-
5	3.21304	0.522	-

* Statistically significant values.

**Table 2:** Results of the $\chi^{2}$ test.

Question	$\chi^{2}$	*p*	*C*
1	13.6230	0.0086 *	0.80
2	23.1366	0.0001 *	0.92
3	5.75845	0.2179	-
4	16.9243	0.0019 *	0.86
5	2.83110	0.5865	-

* Statistically significant values.

**Table 3:** Results of the $\chi^{2}$ test.

Question	$\chi^{2}$	*p*	*C*
1	18.8247	0.0008 *	0.88
2	14.8806	0.0049 *	0.82
3	13.3729	0.0096 *	0.80
4	10.9845	0.0267 *	0.73
5	12.2595	0.0155*	0.77

* Statistically significant values.

**Table 4:** Test results.

Question	$\chi^{2}$	*p*	*C*
1	23.2927	0.0001 *	0.92
2	3.96601	0.4016	-
3	2.95743	0.5649	-
4	19.8137	0.0005 *	0.89
5	13.1354	0.0106 *	0.79

* Statistically significant values.

## Data Availability

Not applicable.

## Appendix A

Questionnaire items in individual areas of pupil readiness

(A) Causes of adaptation difficulties in the emotional area of school readiness

The respondents’ opinions on the causes of adaptation difficulties in emotional readiness of beginning schoolchildren were expressed in the answers to the questions concerning the insufficient school motivation of children, the requirement of physical proximity, and constant contact with an adult, etc. These were the following questions:

“Do you consider insufficient school motivation to be the cause of adaptation difficulties for first-year pupils?”
“Do you consider the slight distraction of a pupil by external stimuli as the cause of adaptation difficulties in first-year pupils?”
“Do you consider the frequent demand for verbal support and encouraging communication to be the cause of the adaptation difficulties of first-year pupils?”
“Do you consider the requirement of physical proximity, constant contact of an adult to be the cause of adaptation difficulties for first-year pupils?”
“Do you consider rejection, anger, aggression to be the cause of pupils’ adaptation difficulties in the 1st grade?”

(B) Causes of adaptation difficulties in the social area of school readiness

In the social field, these were, for example, questions aimed at not respecting the rules in the classroom, avoiding contact with the teacher. The wording of the questions was as follows:

“Do you consider non-compliance with the rules in the classroom or at school to be the cause of adaptation difficulties for first-year pupils?”
“Do you consider the inability of a pupil to make contact with classmates to be the cause of adaptation difficulties for first-year pupils?”
“Do you consider avoiding contact with the teacher to be the cause of the pupils’ adaptation difficulties in the 1st grade?”
“Do you consider the preference for individual activities over group activities to be the cause of adaptation difficulties for first-grade pupils?”
“Do you consider rare verbal contact to be the cause of pupils’ adaptation difficulties in the 1st grade?”

(C) Causes of adaptation difficulties in the intellectual field of school readiness

To find out teachers’ opinions on the causes of adaptation difficulties in the intellectual field, we asked questions about the low level of schoolchildren’s knowledge of the world and misunderstanding of the teacher’s instructions, etc. We asked the respondents the following questions:

“Do you consider the low level of pupils’ knowledge of the world (knowledge about school, learning activities, about man, about his activities, about nature, about art, knowledge about social norms, etc.) as the cause of adaptation difficulties for 1st graders?”
“Do you consider the requirement of play to be the cause of the adaptation difficulties of the pupils in the 1st grade?”
“Do you consider shortcomings in communication (being able to listen, understand the speaker, express your thoughts, express yourself grammatically correctly, etc.) as the cause of adaptation difficulties for first-grade pupils?”
“Do you consider the lack of attention to be the cause of adaptation difficulties in first-grade pupils?”
“Do you consider the misunderstanding of the teacher’s instructions to be the cause of the pupils’ adaptation difficulties in the 1st grade?”

(D) Causes of adaptation difficulties in the psychomotor area of school readiness

Questions within the causes of adaptation difficulties in the psychomotor area of school readiness concerned e.g., the occurrence of motor unrest in children, low levels of fine and gross motor skills, and more. The questionnaire specifically included the following questions:

“Do you consider deficiencies in fine and gross motor skills to be the cause of adaptation difficulties in first-year students?”
“Do you consider involuntary movements to be the cause of adaptation difficulties in first-year students?”
“Do you consider the increased morbidity of the pupil to be the cause of the pupils’ adaptation difficulties in the 1st year?”
“Do you consider lower working capacity to be the cause of adaptation difficulties in first-year students?”
“Do you consider somatic weakness to be the cause of the pupils’ adaptation difficulties in the 1st year?”
